# New discoveries in the molecular landscape of bladder cancer

**DOI:** 10.12688/f1000research.10031.1

**Published:** 2016-12-19

**Authors:** Roger Li, Woonyoung Choi, J.E. Ferguson 3rd, Michael J. Metcalfe, Ashish M. Kamat

**Affiliations:** 1 Department of Urology, The University of Texas MD Anderson Cancer Center, Houston, TX, USA

**Keywords:** bladder, genomics, carcinoma, sequencing

## Abstract

We are currently on the cusp of exponential growth in the understanding of the molecular landscape of bladder cancer. Emerging data regarding the mutational burden and targetable genomic and protein alterations in bladder cancer have allowed us to tap into treatments directed toward specific molecular characteristics of bladder cancer. In parallel, these developments will enable us to better select patients for existing treatments of bladder cancer in a step toward personalized therapy. The present article reviews select discoveries that have advanced our understanding of bladder cancer and gives a glimpse of the exciting opportunities on the not-so-distant horizon.

## Introduction

Over the last decade, new understandings in molecular carcinogenesis as well as advances in genomic sequencing technologies have ushered in a new era of targeted cancer therapy. Rational therapeutic targets have been identified and new drugs successfully deployed in the treatment of various solid tumors. For example, patients with breast and lung cancers are routinely subjected to genotypic/phenotypic assessment, and specific targeted treatments are available for several permutations ^1, 2^. For bladder cancer, however, prognostication and treatment selection still depend primarily on clinical and pathologic characteristics.

**Figure 1.**
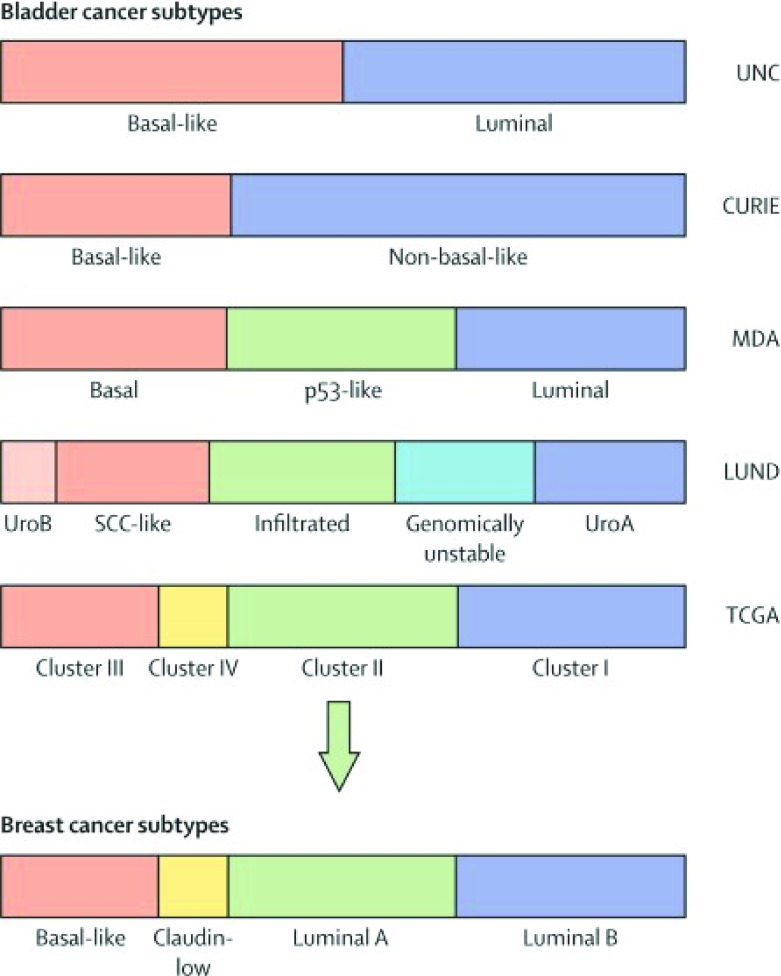
Molecular subtype classification of bladder cancer and breast cancer. Color bars represent subtype classifications made by each institution. Subtype groupings were made independently, and associations were assigned on the basis of the MD Anderson Cancer Center (MDA) classifier. CURIE, Institut Curie; UNC, University of North Carolina. Adapted from Kamat *et al*. ^8^.

In 2014, the comprehensive molecular characterization of muscle-invasive bladder cancer (MIBC) by The Cancer Genome Atlas (TCGA) Research Network produced many new insights into the genetic makeup of MIBC ^3^. Integrated analysis of mRNA, microRNA, and protein expression in 129 muscle-invasive tumors yielded four distinct clusters resembling the intrinsic subtypes identified in breast cancer ^4^. Clusters I and II were similar to luminal A breast cancer and had high mRNA and protein expression of differentiation markers, including GATA3 and FOXA1. Cluster III and IV tumors were similar to basal breast cancer and had high expression of stem/progenitor cytokeratins ^3^. These findings indicate similar pathways of tumorigenesis despite different tissue origins. In addition, these clusters broadly corroborated with the findings of three other contemporary studies ( Figure 1), further validating its accuracy ^5– 8^.

Another significant finding of TCGA was the large number of somatic DNA alterations found in MIBC. Mean and median somatic mutation rates were respectively 7.7 and 5.5 per megabase, more than those found in any adult malignancy other than lung cancer and melanoma ^3^. Importantly, actionable therapeutic targets were found in 69% of the tumors, converging on three main pathways: cell cycle regulation, kinase and phosphatidylinositol-3-OH kinase (PI[3]K) signaling, and chromatin remodeling. Specifically, mutations and somatic copy number alteration were frequently found in histone-modifying genes (89%), components of the SWI/SNF nucleosome remodeling complex (64%), PI(3)K/AKT/mTOR pathway (42%), and the RTK/RAS pathway (44%) ^3^.

Each of these three major findings in TCGA has major implications for the prognostication and treatment design for MIBC. Here, we will review advances in the understanding of MIBC since the landmark publication of TCGA and the new therapeutic strategies they have fostered. Furthermore, the use of high-throughput next-generation sequencing (NGS) has only recently been extended to non-MIBC (NMIBC). Undoubtedly, the experience garnered from studying MIBC using these methods can be readily transferred to help answer many unresolved questions for NMIBC.

## Sensitivity to neoadjuvant chemotherapy

Since the 1980s, cisplatin-based chemotherapy has been recognized as the standard of care for metastatic urothelial carcinoma (UC), specifically with M-VAC (methotrexate, vinblastine, doxorubicin, and cisplatin) established as the superior combination and GC (gemcitabine and cisplatin) as an alternative ^9– 13^. Subsequently, neoadjuvant chemotherapy (NAC) prior to surgical or radiation treatment of MIBC has demonstrated survival benefit in separate phase 3 trials and meta-analyses ^14– 16^. Despite its proven efficacy, NAC rendered only 38% of patients pT0, conferring a modest 5% improvement in 5-year survival. Additionally, toxicity associated with NAC is not insignificant ^14, 15^. In light of this marginal risk-benefit ratio, attempts have been made using clinicopathologic features to refine patient selection for NAC ^17^. However, no clear prediction model for response to NAC exists to date, leading to ongoing debate regarding who should undergo NAC ^18, 19^.

Drawing on the success correlating intrinsic subtypes with NAC response in breast cancer ^20^, Choi *et al*. from MD Anderson Cancer Center used their tripartite molecular subtyping of MIBC (basal, luminal, and p53-like) to predict chemosensitivity ^5^. They found a marked increase in chemoresistance associated with the p53-like tumors compared with the other subtypes. Similar to luminal breast cancers, this chemoresistance was associated with lower levels of proliferation and cell cycle biomarkers in the p53-like subtype ^21^. Interestingly, activation of wild-type p53-like gene expression signature was observed in post-chemotherapy tumor specimens, leading to a larger percentage of p53-like tumors in the post-chemotherapy cohort. In conjunction, these findings suggest that NAC may selectively decimate non-p53-like tumor cells, thus enriching the p53-like gene expression signature that dominates after treatment ^22^. In a subsequent study, the authors showed that basal tumors were associated with better survival outcomes after NAC ^23^. As only M-VAC was used to treat the cohort of tumors analyzed, it is unknown whether this chemoresistant feature of p53-like tumors can be generalized to other therapy combinations.

On the other hand, NGS studies have also linked platinum chemosensitivity to specific genomic alterations. Activating mutations of *ERBB2* (tyrosine kinase receptor) were exclusively found in the pre-treatment tumors resected from patients with complete pathologic response (pT0) ^24^. In addition, mutations in the DNA repair genes are linked with increased chemosensitivity. Mutations in *ERCC2*, a nucleotide excision repair gene, were found to be enriched in patients responding to NAC ^25^. In line with this finding, Plimack *et al*. discovered that alterations in one or more of the DNA repair genes *ATM*, *RB1*, and *FANCC* were enriched in patients downstaged to ≤pT1N0M0 after chemotherapy, leading to improved overall survival ^26^. The authors postulated that the deleterious defects in the DNA repair genes represent a fatal flaw for the associated tumors, making it impossible to recover from the DNA damage incurred by the alkylating agent cisplatin.

Though intriguing, these findings need to be validated in larger studies encompassing diverse patient demographics and chemotherapy regimens. Fortunately, this effort is currently under way, as are efforts to assess the benefit of combining genomic signatures with clinicopathologic characteristics for the prognostication of response to NAC. The knowledge gained will aid in the development of novel chemotherapy/targeted therapy agents in the future.

## Immune checkpoint blockade for metastatic bladder cancer

Overall survival with cisplatin-based chemotherapy, despite its firmly established efficacy in the treatment of metastatic UC, remains rather poor ^27, 28^. The prognosis is especially dismal for patients with relapse after chemotherapy, and median survival ranges from 5 to 7 months ^29^. Moreover, no new drug therapy has been found to be effective in the four decades since the adoption of cisplatin-based chemotherapy. As such, the recent introduction of anti-PD-L1 (programmed death ligand 1) treatment for metastatic UC was met with great enthusiasm ^30, 31^. PD-L1 negatively regulates T-cell function by binding to its receptors programmed death 1 (PD-1 or B7-1) on activated T lymphocytes and other immune cells. The overexpression of PD-L1 in the tumor microenvironment is thought to be the mechanism by which tumor evasion of the host immune system occurs. Blockade of the PD-L1 pathway with a high-affinity engineered human anti-PD-L1 monoclonal immunoglobulin-G1 antibody (atezolizumab) was shown to improve objective response rate in a heavily pre-treated population with poor prognostic features ^32, 33^. Extended median overall survival ranging from 7.9 to 11.4 months was observed. One-year overall survival ranged between 36% and 48% compared with the historic rate of 20% from a pooled analysis ^34^.

Objective response rate, progression-free survival, and overall survival were found to be directly related to PD-L1 expression status on the infiltrating immune cells (ICs). In addition, treatment response correlated with mutation load and was found to be the highest in the luminal tumors subtyped within cluster II of the TCGA scheme ^33^. The finding that PD-L1 was more efficacious in tumors with higher mutation load was consistent with patterns recognized in other malignancies ^35, 36^. Non-synonymous somatic mutations are thought to increase tumor neoantigen burden, leading to increased T-cell recognition and more potent tumoricidal activities unleashed by PD-L1 treatment.

Interestingly, TCGA subtyping was found to have prognostic implications independent of the PD-L1 expression levels in ICs. Despite having lower IC PD-L1 expression, cluster II tumors responded to treatment at a higher rate than the basal cluster III and IV tumors. Their higher response rate to PD-L1 treatment may be attributed to the intrinsic biology of the cluster II tumors. Alternatively, additional immunosuppressive pathways may be used by basal tumors to prevent effective immune activation. Of course, these tumors were previously treated with cisplatinum, which itself has effects of subtype migration as elucidated earlier. Nonetheless, the differential response to PD-L1 therapy in the different tumor subtypes highlights the need for further understanding of their associated immunobiology.

## Targeted therapy: lessons learned and future strategies

Genetic alterations in the mTOR, FGFR, EGFR, and HER2 pathways have long been recognized in subsets of bladder cancer. TCGA and other studies have identified actionable drug targets in over 60% of the tumors interrogated ^3, 37^. Disappointingly, no trial to date has proven efficacy for any rationally designed targeted agent in advanced UC ^38^. The conundrum of futility against the preponderance of potential therapeutic targets can partially be explained by the highly variable genomic landscape of UC uncovered by recent studies. A key finding in a multi-platform analysis of 12 cancer types was the genetic diversity of bladder cancer, splitting into three pan-cancer subtypes ^39^. Such heterogeneity found in advanced UC renders ineffective the one-size-fits-all approach undertaken by most previous trials.

Instead, careful patient selection is needed for targeted drug trials to increase the signal-to-noise ratio derived from effective treatment. Alternatively, in-depth retrospective analysis of exceptional responders may provide insight for refining trial design to yield meaningful outcomes. One such example was illustrated by the phase II study of everolimus in a metastatic UC trial ^40, 41^. Although the trial as a whole failed to achieve the predetermined progression-free survival end point, whole genome sequencing in an exceptional responder revealed mutations that enhanced therapeutic efficacy. Subsequently, by using the newly discovered mutation as a biomarker, the authors were able to demonstrate treatment efficacy in a smaller pre-selected subset ^40^. This prompted the Exceptional Responders Initiative launched by the National Cancer Institute to identify molecular indicators in malignant tissue from exceptional responders using NGS (ClinicalTrials.gov identifier: NCT02243592). With this strategy, afatinib (tyrosine kinase inhibitor of the ErbB receptor family) was found to have efficacy in the subpopulation of patients with platinum-refractory UC with somatic ERBB family alterations ^42^.

Furthermore, the recently published National Comprehensive Cancer Network (NCCN) Bladder Cancer Guidelines recommend broadening the scope of molecular profiling for advanced UC ^43^ in an effort to identify more patients with specific mutations as candidates for various ongoing clinical trials. In addition, those with higher mutational burden or specific tumor subtypes may be selected for immune checkpoint blockade.

## Future outlook

The NGS technology that has brought such major advances in the understanding of MIBC has only recently been used in the study of NMIBC. The traditional dichotomization of bladder cancer into low-grade papillary tumors and invasive carcinoma that arise from flat dysplasia and carcinoma *in situ* ^44^ has been challenged by a proposed subtyping scheme spanning both MIBC and NMIBC ^7^. Others have found NMIBC to exhibit markedly different gene expression profiles than MIBC, leading to its own assigned cluster group on unsupervised hierarchical cluster analysis ^45^.

A recent multi-institutional study of 460 early stage UC identified three major subgroups of NMIBC with basal- and luminal-like characteristics, each associated with different clinicopathologic features and progression-free survival ^46^. However, the authors found imperfect reconciliation between these subtypes to the basal and luminal subtypes found in MIBC. They hypothesized that the three subgroups represented three different developmental pathways of NMIBC.

Further studies using NGS technology in NMIBC are warranted to characterize this group of diverse tumors. Along the way, new insights into the biology of development and progression are likely to be uncovered. Conceivably, biomarkers or subtypes may be identified for susceptibility to intravesical treatment with chemotherapy or bacillus Calmette-Guérin. On the other hand, molecular characteristics of progressive tumor may be collected early on to select patients for early radical treatment to avoid metastatic spread. In fact, evidence is already emerging from transgenic mouse models demonstrating the loss of sonic hedgehog ( *SHH*) expression as an important molecular switch on the path to MIBC ^47^. These are fascinating times both for us in the scientific community and for our patients, and there is hope on the horizon to truly advance the needle in the quest for cure.

## Abbreviations

IC, infiltrating immune cell; MIBC, muscle-invasive bladder cancer; M-VAC, methotrexate, vinblastine, doxorubicin, cisplatin; NAC, neoadjuvant chemotherapy; NGS, next-generation sequencing; NMIBC, non-muscle-invasive bladder cancer; PD-L1, programmed death ligand 1; PI(3)K, phosphatidylinositol-3-OH kinase; TCGA, The Cancer Genome Atlas; UC, urothelial carcinoma.
